# Editorial: Animal Poisoning and Biomarkers of Toxicity

**DOI:** 10.3389/fvets.2022.891483

**Published:** 2022-05-04

**Authors:** Fernando Capela e Silva, Ana Catarina Sousa, Manuel Ramiro Pastorinho, Hazuki Mizukawa, Mayumi Ishizuka

**Affiliations:** ^1^Department of Medical and Health Sciences, School of Health and Human Development, MED-Mediterranean Institute for Agriculture, Environment and Development, University of Évora, Évora, Portugal; ^2^Department of Biology, School of Sciences and Technology, CHRC—Comprehensive Health Research Center, University of Evora, Évora, Portugal; ^3^Department of Medical and Health Sciences, School of Health and Human Development, CHRC—Comprehensive Health Research Center, University of Evora, Évora, Portugal; ^4^Department of Science and Technology for Biological Resources and Environment, Ehime University, Matsuyama, Japan; ^5^Graduate School of Veterinary Medicine, Faculty of Veterinary Medicine, Hokkaido University, Sapporo, Japan

**Keywords:** livestock, pets, wildlife, rodenticides, pesticides, phytopreparations, animal foodstuffs

The worldwide incidence of animal poisoning is unknown. Despite reports pieced together by networks of veterinary clinics and poison control centers, their coverage is usually limited due to widespread under-reporting of cases (1–3). There are several reasons for this reality, being one of the major the wide gaps of knowledge regarding toxicosis agents and their profile (particularly in terms of toxicity mechanisms), since these encompass an extensive variety of synthetic chemicals, molecules of plant and animal origin, as well as drugs (both of use and abuse) (1–3). Together with the lack of specific and sensitive analytical techniques for their detection and quantification, this reality leads to reports being usually submitted in incomplete form, and presented in a case-by case manner (4–6). This situation has created uncertainty when comparing poisoning with other types of clinical findings, such as infectious diseases, traumatic injuries or malignant neoplasms, since toxicosis would come out, at least apparently, as an uncommon cause of disease (3). This has led to appeals for more cooperation and information sharing between countries and institutions (1, 3), even inside “data-rich regions” (7), which typically include North America and Europe. The disproportionately higher volumes of available information in these areas also contribute to introduce geographic biases in available information (2). This reality is perfectly illustrated by European and North American floras, that, despite their disparity in the number of hazardous species (Europe has a much lower number), are both fairly well characterized. Contrastingly, African flora, which is at least as rich in hazardous species as the North American, has been reduced to seemingly anecdotal reports (8, 9). Even in Europe, despite the already mentioned lower number of hazardous species, the number of poisonings (affecting livestock and companion animals) attributed to wild, illicit drug or houseplants (mostly by ingestion) is considerable, since they contain chemical substances in sufficient quantities to cause toxic effects. In the particular case of companion animals, the majority of reported cases refers ingestion of ornamental plants (as opposed to wild), as the source of toxicity, particularly at certain times of the year (8). In addition to plant toxicity, available information indicates that the incidence of animal toxicosis from all causes does not seem to be declining. In the 2010's, a series of reports (10–12) detailed the prevalent toxicants affecting different groups of animals. The authors identified toxic plants and mycotoxins as the most common toxic agents involved in livestock and poultry poisoning, with additional cases being reported for metals (Cu, Pb), pesticides (endosulfan, lindane), and industrial chemicals (e.g., dioxins, polychlorinated biphenyls, dibenzofurans) (11). Regarding companion animals, frequent causes of poisoning include the exposure to anticoagulant rodenticides (coumarins), herbicides (paraquat), and insecticides (organophosphates, carbamate, strychnine, metaldehyde). Also, the inadequate use of human and veterinary pharmaceuticals, and the exposure to household products accounted for a noteworthy part of registered toxicosis reports (10).

Wildlife species are frequently victims of primary (deliberate), but also of secondary poisonings (typically top predators). Metals and metalloids (Pb, Zn, As, Cu, Tl, Cd, and Hg) and pesticides (mostly anticholinergics and anticoagulants) were identified as frequent, often fatal, poisoning agents. In aquatic ecosystems, point or diffuse sources of chemicals and/or from industrial, agricultural, and urban runoff contribute to poisoning incidents. Additionally, chemicals released during environmental catastrophes (which could include any of the previous categories) are a significant cause of poisoning. The type of toxicants described more than two decades ago are, in a large number of cases, not the same, since, at least in the case of synthetic chemicals (including pharmaceuticals) many of them, were banned by legislation. However, new molecules (whose harmlessness is still to be entirely proven) were developed, manufactured in large scales, and started being used in their place. Despite this (partial) change of agents, current reports indicate the continuity of poisoning events toward animals (4, 6, 13–17). Consequently, researchers in this area face the paramount task of unraveling the underlying mechanisms (such as the toxicokinetics and toxicodynamics models and the adverse outcome pathways-AOPs) of new toxicants created by the continuous outpouring of new synthetic chemicals developed for the industry, the agrobusiness and household products market, as well as the growing sector of natural extracts.

Biomarkers can act as indicators or signallers of events occurring in biological systems (18). By permitting the measurement of changes in molecules, biochemical processes, cells, tissues, organs and entire organisms (encompassing physiology, pathology, or behavior) in response to external insult, they provide nuclear knowledge in order to deliver accurate diagnosis under the form of biomarkers of exposure, effects and susceptibility, as well as enabling to delineate therapeutic interventions, and the improvement of key aspects of the drug development process (19). Additionally, they can be noninvasive and can translate between species. In fact, some authors consider that the most valuable are those that can be simultaneously used in animals and humans (19).

In this Research Topic of Frontiers in Veterinary Science/Veterinary Pharmacology and Toxicology, 7 manuscripts were published: 1 Review, 5 original Research Articles and 1 Brief Research Report, whose main results and contributions are briefly presented below.

The Review by Rached et al. approaches the toxicodynamics and toxicokinetics of anticoagulant rodenticides (AR) in various animal species. ARs are widely used, causing inadvertent primary and secondary exposure to non-target domestic and wildlife species through direct ingestion of the baits or by consumption of poisoned prey. The authors provide an overview of different biomarkers applied to characterize and discern the exposure and toxic effects of ARs, highlighting strengths and weaknesses of the different assays (including the metabolomics approach) and calling attention upon the various interpretation and application biases raised by sample collection, storage and processing; additionally possible new biomarkers are described while highlighting their capabilities.

The retrospective study of Grilo et al., presents the toxicological results from domestic species (dog, cat, sheep, cows, and horses), wildlife species (red foxes, birds of prey, lynx, and wild boar), and food baits, realized from January 2014 up until October 2020, in Portugal. This study allowed to realize that a great number of positive samples involved banned pesticides (i.e., Aldicarb and Strychnine) but, at the same time, many positive cases were due to the exposure to commercially available products (i.e., Methiocarb and Anticoagulant rodenticides). The areas where domestic species are the most affected (i.e., Setubal and Lisbon) and the areas where the wild animals are the mainly affected species (i.e., Faro, Castelo Branco, and Braganza), were also identified.

Plants produce a wide variety of metabolites, which in many cases are toxic and that can cause metabolic changes harmful to animals, but in some cases, can help to counteract some of the toxic effects of other agents/xenobiotics. In this sense, the other five manuscripts of this Research Topic studied some metabolites from different plants and their effects, potential or real/effective, on different animal species.

In their study, Hu et al., using a bioactivity-guided approach, investigated in mice, the toxic ingredients of *Macleaya cordata*, a perennial herb known for a wide range of pharmacological activities. The results indicate that protopine (a major bioactive constituents of multiple phytopreparations applied in veterinary and human medicine, and the primary toxic constituent in *M. cordata*) might pose a serious health threat to humans and animals.

The presence of toxicants and bioactive substances in animal foodstuffs is a motive of concern in animal welfare, particularly because a large number of precise molecular mechanisms (and their interaction), remain unknown. Such is the case of copper overload and nephrotoxicity. The study by Peng et al., investigated the molecular mechanism of copper sulfate (CuSO_4_)-induced nephrotoxicity and the protective effect of the natural compound quercetin using a mouse model. Serum biomarkers, oxidative stress biomarkers, changes in histopathology and gene and protein expression were examined in blood and kidneys. It was shown that quercetin, by inhibiting mitochondrial apoptotic and NF-κB pathways and by activating the Nrf2/HO-1 pathway, was able to reduce oxidative stress, apoptosis and inflammatory responses. As such, quercetin appears to be a promising attenuating agent against CuSO_4_-induced nephrotoxicity.

In another study Chicoine et al. used healthy Beagle-cross dogs to evaluate the pharmacokinetics and safety of various oral doses of a Cannabis herbal extract (CHE) containing a 1:20 ratio of Δ9-tetrahydrocannabinol (THC):cannabidiol (CBD). The authors' consensus is that the limited incidence and severity of adverse events observed in the low and medium CHE dose groups would be considered an acceptable risk by most dog owners the same not happening for the higher doses due to clinically relevant neurological signs observed. In this way and considering non-proportional increases in plasma cannabinoid concentrations with increasing doses, as well as potential differences in CHE product composition and bioavailability, it is suggested that Veterinarians should actively counsel owners electing to administer these products.

Laminitis, a disease that affects the feet of ungulates and found mostly in horses and cattle. Consists in the failure of the dermal-epidermal interface of the foot, causing, at the early phases of the disease, the release of myeloperoxidase (a pro-oxidant enzyme present in activated neutrophils), in plasma, skin, and laminar tissue. Mouithys-Mickalad et al., conducted a study where, by the oral administration of a black walnut extract, laminitis was induced in horses. Black walnuts contain juglone (a naphthoquinone derivative endowed with redox properties), which has been implicated in the activation of neutrophils. However, the results indicate that juglone is not the activation factor for equine laminitis if the motive is the modulation of neutrophil activation.

Finally in a Brief Research Report, Câmara et al. present a study whose objective was to identify important prognostic parameters that can determine the severity of spontaneous poisoning by *Crotalaria spectabilis* in horses. At the end of the 12-months study, only 30 of the 42 animals that started the study survived and the analysis of blood samples from all the horses, spontaneously poisoned by oats contaminated with *C. spectabilis* seeds, had higher levels of several biomarkers than the reference values. The authors concluded that serum γ-glutamyl transferase activity and direct bilirubin concentration may be useful prognostic indicators for assessing the severity of *C. spectabilis*-poisoned horses.

The applicability of biomarkers in a wide range of fields, spanning disciplines as different as risk assessment, environmental regulation, and Veterinary and Human Public Health, conveys the very important role they possess in multidisciplinary research, and the necessity of continued study. The health and wellbeing of humans, domestic animals and wild species is profoundly intertwined as the eloquent and dramatic cases of Minamata Disease [when “dancing cats” presented an unheeded early warning (20)], the Bhopal Disaster [where thousands of men, women, children, domestic animals and wildlife died abruptly side by side (2)] and the collapse of the World Trade Center in New York City [in which aftermath “Ground Zero illnesses” equally affected first responders and their working dogs (21)] illustrate. In the face of this and many other evidence, we share the concept that “the convergence of people, animals, and our environment has created a new dynamic in which the health of each group is inextricably interconnected” (22) and it is our opinion that, although the impact of toxic agents on wildlife, companion animals, and human health has historically been addressed separately, an extensive application of biomarkers within the scope of the One *Health concept*, more than presenting interesting and relevant intellectual challenges, would provide fundamental contributes to the full understanding of common poisoning susceptibilities among all living species.

## Author Contributions

FCS, AS, and MP written the draft being this amended and revised by HM and MI. All authors contributed to the article and approved the submitted version.

## Conflict of Interest

The authors declare that the research was conducted in the absence of any commercial or financial relationships that could be construed as a potential conflict of interest.

## Publisher's Note

All claims expressed in this article are solely those of the authors and do not necessarily represent those of their affiliated organizations, or those of the publisher, the editors and the reviewers. Any product that may be evaluated in this article, or claim that may be made by its manufacturer, is not guaranteed or endorsed by the publisher.
